# Reply: Patterns of nodal recurrence after omission of elective nodal irradiation for limited-stage small-cell lung cancer

**DOI:** 10.1038/sj.bjc.6603861

**Published:** 2007-06-26

**Authors:** J Belderbos, P Baas, S Senan

**Affiliations:** 1 Department of Radiation Oncology, Antoni van Leeuwenhoekhuis/Netherlands Cancer Institute, Amsterdam, The Netherlands; 2 Department of Thorax Oncology, Antoni van Leeuwenhoekhuis/Netherlands Cancer Institute, Amsterdam, The Netherlands; 3 Department of Radiation Oncology, VU University Medical Center, Amsterdam, The Netherlands

**Sir**,

We thank Dr Chun-Ru Chien *et al* for their interest in our recent study and for their remarks concerning the safety of using involved-field irradiation in treating limited-stage small-cell lung cancer (SCLC). We have updated our analysis of failure patterns in patients with limited-stage SCLC who were treated in our phase II study of involved-field irradiation during the second and third cycles of carboplatin, paclitaxel and etoposide (Baas *et al*, 2006). Of the 36 patients, isolated out-of-field nodal failures were only seen in two patients, one of whom developed an ipsilateral supraclavicular nodal recurrence after 10 months and the other developed a recurrence in the left hilar region after 18 months follow-up. In-field recurrences were observed in nine patients, and this was the sole site of recurrence in four patients. One patient developed a large-cell carcinoma within the treatment field 5.2 years after the diagnosis of SCLC. In two other patients, simultaneous recurrences manifested both within- and out-of-field regional nodes. A further two patients presented with a simultaneous local recurrence and distant metastases.

Recently, De Ruysscher *et al* (2006)published their prospective evaluation of patterns of recurrence in twenty-seven limited-stage SCLC patients irradiated without elective nodal fields (without use of 18-fluoro-2-deoxyglucose positron-emission tomography (18FDG-PET) scans). Three of their 27 patients developed an isolated nodal failure in the ipsilateral supraclavicular region. They concluded that the omission of elective nodal irradiation in SCLC patients should be performed in the context of trials only. It could be argued that the dose used in our trial of 45 Gy in 5 weeks was too low, and local recurrences occurred before nodal failures became evident. De Ruysscher prescribed a more intense radiotherapy schedule of 45 Gy given in 3 weeks (twice daily irradiation with a 6–8 h interval). It is unclear as to why the supraclavicular region was prone to isolated recurrences. In general, this area is difficult to assess on the computed tomography (CT) scan of the thorax (and is often not fully accessed). A solution for this problem is to ensure an optimal contrast-enhanced CT scan, which includes the supraclavicular region. The addition of a routine ultrasound of the supraclavicular area has been shown to improve staging in both NSCLC (non-small-cell lungs cancer) and SCLC (Van Overhagen *et al*, 2004). In addition, recent literature suggests that 18FDG-PET scans could be of additional value in staging patients with limited-stage SCLC (Bradley *et al*, 2004; Fischer *et al*, 2007).

A phase III trial of concurrent chemoradiotherapy without elective nodal irradiation in limited-stage SCLC, where patients will be randomised to either once daily irradiation to a dose of 66 Gy or twice daily irradiation to a dose of 45 Gy will shortly commence (Faivre-Finn, 2006). Such a trial will generate more reliable data on the safety of omitting elective nodal irradiation in this disease. Until additional data become available, we would suggest that optimal nodal staging that incorporates an ultrasound examination of the neck, and possibly use of 18FDG-PET scan should be performed for patients undergoing involved-field radiotherapy for limited-stage SCLC.
